# Potential Application of *Hippophae Rhamnoides* in Wheat Bread Production

**DOI:** 10.3390/molecules25061272

**Published:** 2020-03-11

**Authors:** Aliona Ghendov-Mosanu, Elena Cristea, Antoanela Patras, Rodica Sturza, Silvica Padureanu, Olga Deseatnicova, Nadejda Turculet, Olga Boestean, Marius Niculaua

**Affiliations:** 1 Technical University of Moldova; 9/9 Studentilor St, MD-2045 Chisinau, Republic of Moldova; 2 “Ion Ionescu de la Brad” University of Agricultural Sciences and Veterinary Medicine of Iasi, Romania, 3 Mihail Sadoveanu Alley, 700490, Iasi, Romania; 3 Research Center for Oenology, Romanian Academy, Iasi Branch, 9 Mihail Sadoveanu Alley, 700490 Iasi, Romania

**Keywords:** sea buckthorn berries, bioactive compounds, polyphenols, natural additive, antioxidant, antimicrobial, shelf life, organoleptic properties

## Abstract

Sea buckthorn (*Hippophae rhamnoides*) berries are well known for their content in bioactive compounds, high acidity, bright yellow color, pleasant taste and odor, thus their addition in a basic food such as bread could be an opportunity for modern food producers. The aim of the present research was to investigate the characteristics and the effects of the berry’ flour added in wheat bread (in concentration of 1%, 3% and 5%) on sensory, physicochemical and antioxidant properties, and also bread shelf life. Berry flour contained total polyphenols—1467 mg gallic acid equivalents (GAE)/100 g, of which flavonoids—555 mg GAE/100 g, cinnamic acids—425 mg caffeic acid equivalents (CAE)/100 g, flavonols—668 mg quercetin equivalents (QE)/100 g. The main identified phenolics were catechin, hyperoside, chlorogenic acid, cis- and trans-resveratrol, ferulic and protocatechuic acids, procyanidins B1 and B2, epicatechin, gallic acid, quercetin, *p*- and *m*-hydroxybenzoic acids. The antioxidant activity was 7.64 mmol TE/100 g, and carotenoids content 34.93 ± 1.3 mg/100 g. The addition of berry flour increased the antioxidant activity of bread and the shelf life up to 120 h by inhibiting the development of rope spoilage. The obtained results recommend the addition of 1% *Hippophae rhamnoides* berry flour in wheat bread, in order to obtain a product enriched in health-promoting biomolecules, with better sensorial and antioxidant properties and longer shelf life.

## 1. Introduction

For millennia, bread and other bakery products have been and currently still are staples in many countries. Although bread making may look simple at first glance, its production is becoming increasingly complex due to consumer requirements regarding functionality and nutritional properties, but also in terms of sensory characteristics. These products represent an important part of the foods produced in many countries, e.g., the average volume index of bakery and flour products consumed in the Republic of Moldova between 2012 and 2017 was 102.6 [1].

At present, food producers pay much attention to food safety. In bakery products, this aspect is related to the quality of wheat flour, specifically wheat grain contamination by microorganisms. In recent years, flour and bakery products have been increasingly affected by rope spoilage [2]. Generated by a relatively heterogeneous microbial population of bacteria belonging to the genus *Bacillus*, e.g., *Bacillus subtilis* and *Bacillus mesentericus,* this is a serious problem for manufacturers [3,4]. A potential solution to this issue resides in a direct correlation between the content of bioactive compounds of certain unusual natural food additives/ingredients that may be used and its antibacterial potential [5].

Well known for their content in antioxidants (ascorbic acid, polyphenols, carotenoids), high acidity, bright yellow color, pleasant taste and odor, sea buckthorn (*Hippophae rhamnoides*) berries could be added to the recipe of different foods [6], including bread. It is expected, except the input of bioactive compounds, to also improve sensorial properties, antioxidant potential, microbiological stability and shelf life of resulting products. The sea buckthorn antimicrobial properties against *Klebsiella pneumoniae, Salmonella enterica, Pseudomonas aeruginosa, Acinetobacter baumannii, Proteus mirabilis,* methicillin-resistant *Staphylococcus aureus, Enterococcus faecalis, Enterococcus faecium, Bacillus cereus, Escherichia coli* were stated by previous studies [7,8].

The purpose of this research was to study the effects of different concentrations of sea buckthorn berry flour on the sensory, physicochemical and antioxidant properties, as well as shelf life of the wheat bread, in order to diversify the range of bakery products.

## 2. Results and Discussion

The mass of 100 fresh sea buckthorn fruits was 72 ± 10 g, with 18.1 ± 0.11% dry weight, 8.94 ± 0.15° Brix soluble solids in fruit pulps, and total titratable acidity of 1.7 ± 0.1 g citric acid/100 g fresh weight.

### 2.1. Characteristics of Wheat Flour and Sea Buckthorn Berry Flour

The physicochemical quality indicators of the wheat flour and the concentrations of polyphenols and the antioxidant activity of the berry flour are represented in Table 1 and Table 2, respectively.

In our study, white wheat flour without grain husk (which was separated from flour after milling, in the form of bran) was used. It is known that white wheat flour has very low phenolic content, since the polyphenols are present especially in the removed bran. Phenolic acids and flavonoids are the most common forms of phenolic compounds in whole wheat. They are found mainly in grain husk, as soluble free compounds, soluble conjugates that are esterified to sugars and other low molecular mass compounds, and in the insoluble bound form linked through ester bonds to cell wall structural components such as cellulose, lignin, and proteins [9].

Concerning the berry flour, the results for total polyphenols revealed 1467 mg gallic acid equivalents (GAE)/100 g, when determined by the Folin-Ciocalteu method and 1311 mg GAE/100 g, when determined by absorbance at 280 nm. Among them, 555 mg GAE/100 g were flavonoids. These differences can be explained by the interferences which occur when employing the Folin-Ciocalteu method, which basically quantifies the reducing potential of a solution and, therefore, overestimates the total polyphenol content [10].

The main seven individual phenolics identified in the sea buckthorn berry flour were catechin (35.3 mg/100 g), hyperoside (23.6 mg/100 g), chlorogenic acid (11.1 mg/100 g), trans- and cis-resveratrol (10.4 mg/100 g and 10.8 mg/100 g, respectively), ferulic acid (10.3 mg/100 g) and protocatechuic acid (7.0 mg/100 g).

The study of Hajazimi et al. [11] evaluated and applied a HPLC-CoulArray method (high-performance liquid chromatography with coulometric array detection) for simultaneous determination of flavonols and phenolic acids in several berries including sea buckthorn. The aforementioned authors found quercetin, myricetin, isorhametic, caffeic, ferulic, and *p*-coumaric acids in an extract prepared with 50% aqueous methanol containing TBHQ (tert-butylhydroquinone) antioxidant and 1.2 M HCl. But, contrary to our results, they didn’t identify gallic and vanillic acids. The total concentration of polyphenols determined in sea buckthorn berries was 270.5 mg/100 g dry weight [11], which is significantly lower compared to the amount found in the current research. Ma et al. [12] found twenty-six flavonol glycosides in wild sea buckthorn varieties from China, Finland and Canada. Quercetin was also one of the major aglycones identified in the respective study, while Arimboor et al. [13] showed that gallic acid was the predominant phenolic acid in both sea buckthorn berries and leaves, among gallic, protocatechuic, *p*-hydroxybenzoic, vanillic, salicylic, *p*-coumaric, cinnamic, caffeic and ferulic acids. The present study showed that ferulic acid is the most abundant phenolic acid. Geographical origin, climate, soil, harvest time, genetic and cultivar variability have a great effect on both type and content of the identified polyphenols [14,15], thus such differences are not surprising. Ten sea buckthorn populations from different natural habitats located in the Central Albraz Mountains in Iran were collected and evaluated in 2014 and 2015 [15]. The highest fruit flavonoid content in 2014 and 2015, i.e., 2.40 and 3.19 mg/g was reported for Baladeh populations, while the lowest, i.e., 1.04 and 0.92 mg/g—for the Dehdar population. The same authors have documented that flavonoid content varies significantly among genotypes from Russia, Canada, China, and Finland [15,16].

Ma et al. [12] and Burri et al. [17] reported that antioxidant capacity and the content of polyphenols depend significantly on the species and the cultivar in question. The method used to determine this parameter can also impact greatly the result, as presented in Table 2 (total polyphenols).

Kant et al. [18] have analyzed the content of polyphenols and the antioxidant activity using various methods (among which ABTS (2,2’-azino-bis(3-ethylbenzothiazoline-6-sulphonic acid)) radical scavenging), of Indian sea buckthorn extracts obtained in various solvents, i.e., methanolic and aqueous. The extracts were able to scavenge different radicals in a concentration dependent manner. The linear regression analysis showed that 100% methanolic extract was better scavenger of ABTS, DPPH (2,2-diphenyl-1-picrylhydrazyl) and hydroxyl radicals. Therefore, both the extraction technique and the solvent influence the results for total polyphenols [18].

A relatively low amount of carotenoids of 34.93 ± 1.3 mg/100 g was found in sea buckthorn berry flour (Table 2), while, the comparison of the abovementioned result with those published by Pop et al. [19] showed slight differences. The authors obtained values comprised between 53 and 97 mg/100 g dry weight in berries of six varieties of Carpathian sea buckthorn (*Hippophae rhamnoides* L., ssp. *Carpatica*) analyzed using a combination of the HPLC-PAD (high-performance liquid chromatography with photodiode array detector), GC-MS (gas chromatography–mass spectrometry) and UHPLC-PAD-ESI-MS (ultra-high performance liquid chromatography-photodiode array detector electrospray ionization-mass spectrometry) techniques [19]. Andersson et al. [20] report contents comprised between 12 mg/100 g dry weight and 142.5 mg/100 g dry weight after analyzing berries from four cultivars of sea buckthorn during ripening in three consecutive years. A study of the carotenoids’ composition in sea buckthorn berry flour is therefore recommended, especially considering that carotenoids also exhibit antibacterial properties [21,22].

### 2.2. Effects of Sea Buckthorn Berry Flour Adition on Wheat Bread’s Properties

The results of sensory analysis of the wheat bread samples with added sea buckthorn berry flour are presented in Table 3.

The results of the sensory analysis have shown that the addition of 1% sea buckthorn concentration influenced favorably the organoleptic index of the sample obtained. This sample had a smooth, glossy, golden crust, elastic core, dry taste, well-developed porosity, pleasant taste and aroma. On the other hand, samples with 3% and 5% sea buckthorn berry flour (SBBF) had dark crust, dry crumb, poorly developed porosity, and a specific sea buckthorn berry flavor and odor. Nevertheless, the total scores on the organoleptic analysis of the samples containing 3% and 5% SBBF are within the 24.1–30.0 interval, which implies that the products are of very good quality [23].

The physicochemical quality indicators of the products obtained with the addition of sea buckthorn berry flour and control are presented in Table 4.

The analysis of the results presented in Table 4 has shown that the addition of sea buckthorn berry flour influenced the moisture content of the bread core. Therefore, sea buckthorn slows down the aging process due to the ability of its components (cellulose, hemicellulose, pectin) to bind and retain water in the product. The slow migration of moisture during the storage of the bakery product contributes to maintaining the freshness of the bread core [24].

The acidity of the bakery products increased with the concentration of the added sea buckthorn berry flour. This phenomenon can be explained by the presence of organic acids and sugars from sea buckthorn berry flour, which accelerated dough fermentation. Consequently, the acidity of the samples with sea buckthorn increased by 100–291.7% compared to control.

Porosity plays a leading role in the digestibility of bakery products. The higher the porosity of the bread core, the easier to digest it by the consumer’s body [25]. According to our research, the porosity of the core in the sample with the addition of 1% SBBF increased to 72.7%. Vitamins and simple carbohydrates of sea buckthorn stimulate the fermentative activity of bakery yeast, influencing the porosity of the bread core. The porosity decreased by 5.7% and 17.4% in the samples with 3% and 5% sea buckthorn, respectively, compared to control. This can be explained by the fact that the obtained dough had a low extensibility, contributing to the lowering of the gas retention capacity during fermentation. As a result, the specific volume of the respective samples was decreased by 17.7% and 23.6% compared to control. In the case of 1% SBBF, the increase of specific volume was by 1% compared to control.

The influence of the addition of sea buckthorn on the microbiological safety of wheat bread was investigated (Table 5). The analysis of the results showed that even 1% of added sea buckthorn berry flour reduced the risk of rope spoilage in wheat bread. The spoilage appeared after 96 h for 1% SBBF, after 120 h for 3% SBBF and after 144 h for 5% SBBF in bread, compared to the control, for which spoilage appeared after 72h. The shelf life of the fortified products was extended by 24, 48 and, 72 h, respectively.

The increasing time of storage in the presence of berry flour may be connected to the significant content of phenolics that we noticed, as many studies document the antimicrobial effect of the polyphenols and recommend the commercial use of polyphenol rich extracts in food processing [26,27,28,29]. Sea buckthorn berries contain a series of biologically active substances, such as polyphenols and other natural antioxidants, which inhibit the development of microorganisms and allow the stabilization of the food matrix [30].

It is well known that adding rich sources of antioxidants of plant origin influences not only the microbiological stability of food, but also the antioxidant activity [31,32].

**Figure 1:**
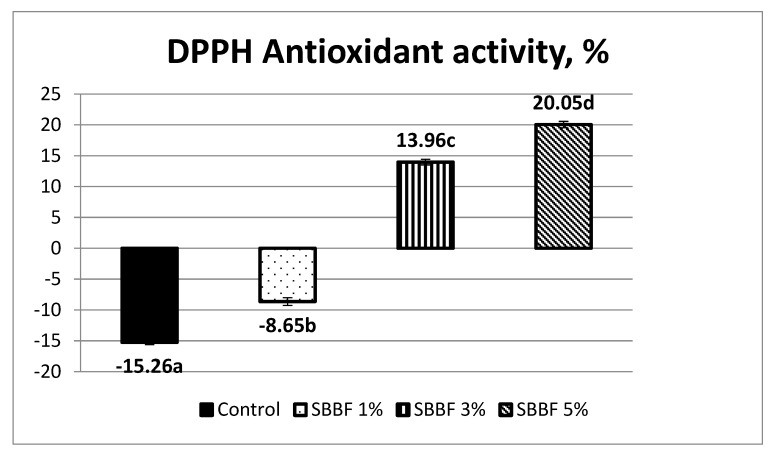
DPPH antioxidant activity (%) of the wheat bread samples made with and without sea buckthorn berry flour (error bars represent standard deviation, different letters indicate significantly different results).

The control sample had a negative value of the DPPH antioxidant activity, i.e., −15.26 ± 0.36% (Figure 1). This can be explained by the fact that in the experimental conditions of gastric digestion simulation (acid pH), the starch of wheat bread breaks down into glucose. According to Pilar de Torre et al. (2019), glucose exhibits a prooxidant effect [33].

The antioxidant activity was improved by a slightly increase, but remained negative (−8.65 ± 0.62%), when 1% sea buckthorn berry flour was added. Adding 3% SBBF provided a positive value (13.96 ± 0.45). For samples with 5% SBBF, an appreciable antiradical activity (20.05 ± 0.51%) was noticed. The explanation is that in the SBBF 1% bread, even if antioxidant properties are improved compared to control, the prooxidant effect of the glucose still prevails, while at higher concentrations of added sea buckthorn berry flour (SBBF 3% and SBBF 5%), the antioxidant effects of the bioactive compounds originated from sea buckthorn exceeds the mentioned prooxidant ones, and consequently, the antioxidant activity has positive values and increases when the SBBF percent in bread increases (Figure 1).

Thus, the use of sea buckthorn berry flour at low concentration (1%) in wheat flour products positively influences the structural-mechanical, physicochemical, organoleptic, antioxidant properties and microbiological stability of the finished products.

**Figure 2:**
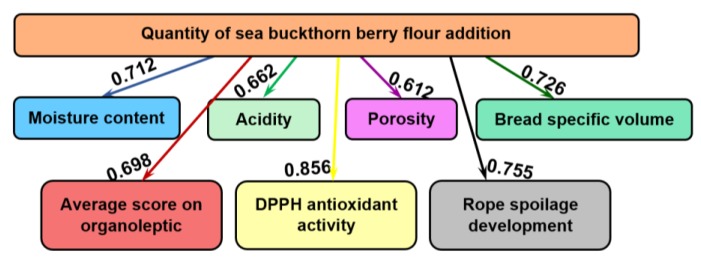
The informational analysis regarding the influence of various quantities of sea buckthorn berry flour on wheat bread physicochemical and organoleptic parameters, DPPH antioxidant activity and rope spoilage development.

Figure 2 summarizes the influences exhibited by the addition of various amounts of sea buckthorn berry flour on the physicochemical indicators (moisture content, acidity, porosity, bread specific volume), the total score of the organoleptic indices, the in vitro DPPH antioxidant activity and the development of rope spoilage in wheat bread, i.e., the appearance of the first signs of disease. The values of the mutual influence are presented on the arrows.

It was shown that the added amounts of sea buckthorn berry flour influence greatly (0.856 bits) DPPH antioxidant activity. On a decreasing scale, the next influence is on the development of rope spoilage in bread, 0.755 bits. The third influence in size is on the specific volume of bread, with mutual information of 0.726 bits and the smallest influence has been exhibited on the porosity of bread, 0.612 bits.

## 3. Materials and Methods

D(-)-quinic acid 98%, sinapic acid (98%), ABTS (2,2′-azino-bis(3-ethylbenzothiazoline-6-sulphonic acid), methyl 4-hydroxy-3-methoxycinnamate (99%) were purchased from Alfa Aesar (Germany), Folin–Ciocalteu reagent from Merck (Germany), (+)-catechin 98%, caffeic acid, syringic acid, ferulic acid, gallic acid (98%), protocatechuic acid, gentisic acid, parahydroxybenzoic acid, salicylic acid (99.9%), para-coumaric acid were provided by Sigma (Germany, Japan, China). Procyanidin B1, procyanidin B2, polydatin, hyperoside came from Extrasynthese (France). Trans-resveratrol was purchased from TCI Europe (Belgium). DPPH (2,2-diphenyl-1-picrylhydrazyl) and quercetin (>95%) from Sigma-Aldrich.

The sea buckthorn berry flour used for the research was prepared from “Clara” variety berries harvested from a plantation in Pohrebea village in Dubăsari district situated in the centre of the Republic of Moldova. The plantation is located on flat land at 47°10′34″ latitude, 29°10′4″ longitude and 23 m altitude above sea level.

The dry weight, soluble solids and titratable acidity of fresh fruits were determined using the methods described in ISI Handbook of Food Analysis [34], ISO 2173:2003 [35], and ISO 750:1998 [36], respectively. In producing the flour, the berries were dried by convective method at the temperature of 65 ± 1 °C, ground to powder and sieved. The granularity of the finished product was 40 ± 10 µm.

A sample of high quality wheat flour from Moldova was used for testing. The quality indicators of the flour, namely moisture, ash content, wet gluten content, acidity were analyzed according to standard international methods [37].

### 3.1. Extraction

The sea buckthorn berry flour was used to prepare a 50% hydroethanolic extract at room temperature. The solid:liquid ratio was 1:10 and mechanical stirring 60 rpm for 30 min was employed to maximize extraction. The obtained extract was stored in glass bottles at t = 4.0 ± 1.0 °C, in the dark prior to analysis.

### 3.2. Antioxidant Activity of the Sea Buckthorn Berry Flour by Reaction with ABTS (2,2′-Azino-Bis(3-Ethylbenzothiazoline-6-Sulphonic Acid)) Radical

The antioxidant activity of the sea buckthorn berry flour was measured following the method described by Re at al. [38], which employs ABTS radical. The results were expressed as mmol trolox equivalents per 100 g berry powder (mmol TE/100 g) from a calibration curve (0–2000 μmol/L).

### 3.3. Total Polyphenols and Flavonoids in Sea Buckthorn Berry Flour by Folin-Ciocalteu

The content of total polyphenols was determined following the method described by Singleton and Rossi [39] and the results were calculated from a calibration curve using gallic acid (0–500 mg/L) and expressed in equivalents of gallic acid per 100 g berry powder (mg GAE/100 g).

The total flavonoids’ content was determined using the precipitation with formaldehyde, followed by Folin-Ciocalteu reaction, according to the method described by Spranger et al. [40].

### 3.4. Total Polyphenols in Sea Buckthorn Berry Flour by Absorbance at 280

Another tool used to determine total polyphenols’ content was the method described by Ribereau-Gayon et al. [41] which employs absorption at 280 nm. The results were expressed as mg equivalent of gallic acid per 100 g berry powder (mg GAE/100 g) from a calibration curve (0–50 mg/L).

### 3.5. Total Cinnamic Acids in Sea Buckthorn Berry Flour

The total cinnamic acids content was assessed using the method described by Demir et al. [42]. The results were expressed as mg caffeic acid equivalents per 100 g berry powder (mg CAE/100 g) based on a calibration curve (0–50 mg/L) with standard of caffeic acid.

### 3.6. Total Flavonols in Sea Buckthorn Berry Flour

Total flavonols were determined using the method described by Demir et al. [42]. The results were expressed as mg quercetin equivalents per 100 g berry powder (mg QE/100 g) based on a calibration curve (0–50 mg/L, R^2^ = 0.9967) with standard of quercetin.

### 3.7. Total Carotenoids in Sea Buckthorn Berry Flour

The total content of carotenoids was determined following the modified method described by Pop et al. [43].

The plant material (3 g) was extracted with a mixture of methanol/ethyl acetate/petroleum ether (1: 1: 1, *v*/*v*/*v*). After filtering the extract, the residue was re-extracted twice using the same solvent mixture. Total carotenoid content was determined using the spectrophotometric method with an UV-VIS spectrophotometer. The absorption spectrum was plotted and the total carotenoid content was measured at the maximum absorbance wavelength (λmax. = 450 nm). The experiments were done under subdued light.

### 3.8. The Analysis of Polyphenols in Sea Buckthorn Berry Flour by HPLC

The content of individual phenolics was analyzed using the Agilent 1100 Series HPLC following the method described by Cristea et al. [44].

### 3.9. The Analysis of L-ascorbic Acid in Sea Buckthorn Berry Flour by HPLC

A Shimadzu 2010 HPLC system equipped with PDA detector and Grace Alltima C18 column (100 × 3 mm, 3 µm) was used to determine L-ascorbic acid content in sea buckthorn berry flour. The elution gradient used 15 mM phosphate buffer at pH 2.7 (A) and methanol (B) as a mobile phase with a flow rate of 0.4 mL/min. The gradient was achieved as follows: min 0: 10% B, min 5: 20% B, min 10: 10% B. The column temperature was 30 °C and the volume injected, 20 μL. UV wavelength for L-ascorbic acid detection was 244 nm, and the retention time determined was 1.85 min. The results of the total vitamin C content, expressed in mg/100 g, were obtained using the calibration curve of L-ascorbic acid (0.3–1 mg/L, R^2^ = 0.999) [45].

### 3.10. Bread Making

Samples of wheat bread were made using 1% (1% SBBF), 3% (3% SBBF), and 5% (5% SBBF) sea buckthorn berry flour to determine its influence on organoleptic, physicochemical and microbiological indicators of bread. The amount of SBBF replaced an equal amount of wheat flour. The control sample was prepared without adding any sea buckthorn material. Wheat flour of superior quality from SC Beatrice-Com SRL, R. Moldova, compressed yeast *Saccharomyces cerevisiae* Pakmaya from Rompak SRL, Bunetto iodized salt from Romania, and water were used in the bread making. The wheat bread samples were baked from dough prepared by direct method, following the requirements of GOST 27669-88 [46]. The dough preparation process was single-phase with the fermentation time of 3 h at 31 ± 1 °C. The obtained samples were analyzed 12 h after baking to determine the influence of sea buckthorn berry flour on the quality of bakery products. Moisture content, acidity, porosity and bread specific volume were tested following standard method SR 91:2007 [47].

### 3.11. Antioxidant Activity of the Sea Buckthorn Berry Flour and Bread by DPPH (2,2-Diphenyl-1-Picrylhydrazyl) Radical

The antiradical DPPH activity of the bread samples was determined in vitro under gastric digestion simulation in the presence of pepsin (150 mg/100g of product), at pH = 2.0 ± 0.1 (1.5 M HCl), at t = 37.0 ± 0.1 °C in a water bath, under agitation at 60 rpm for 2 h [48]. The samples were then centrifuged at 6000 rpm for 10 min at 20 ± 1 °C, filtered and tested following the method described by Brand-Williams et al. [49]. The results were expressed according to the formula:

(1) $$AA(\%)\;=\;\frac{A_{0}-A_{30}}{A_{0}}\;\cdot \;100\;\left(\%\right),$$

where, *A*_0_—the initial absorbance of the DPPH^•^ solution and *A*_30_—the absorbance of the DPPH^•^ solution in presence of the studied extract, after 30 min from the addition of the extract.

### 3.12. Sensory Analysis of the Wheat Bread Samples

The sensory evaluation of the products obtained was performed using a 30-point scale method described by Lawless and Heymann [23]. The bread receiving between 24.1 and 30 points is considered of very good quality; between 18.1 and 24.0 points is good quality; between 12.1 and 18.0 points is satisfactory quality; between 6.1 and 12.0 is bad quality; between 0.1 and 6.0 is very bad quality. The shape, the crust surface, the color, the appearance in section, the crumb consistency, the taste, and the odor were assessed by a trained panel of tasters. For the quantification of the sensory characteristics were used the criteria from Table 6.

### 3.13. The Study of Rope Spoilage Development in Bread Samples

The development of rope spoilage in the wheat bread samples was analyzed following the method of Thompson et al. [50].

### 3.14. Statistical Analysis

The mean values and the standard deviations of the sea buckthorn berry flour parameters were calculated from three parallel extraction experiments. All other tests were also performed in triplicate, at room temperature t = 20 ± 1 °C. One-way ANOVA and post-hoc Tukey test were used to distinguish between means. The considered significance level was *p* ≤ 0.05. All calculations were made using IBM SPSS Statistics 23 and Microsoft Excel 2010. The information analysis was done using MathWorks (Inc., Natick, MA, USA), which allows the evaluation of the mutual influences among determined parameters. Based on two concepts, i.e., entropy and information, it uses the bit as unit of measure. As a result, more certain predictions can be made when the values of the mutual information are higher, and thus the uncertainties lower [51,52].

## 4. Conclusions

The addition of sea buckthorn berry flour in wheat bread prolonged its shelf life by 24–72 h and improved the antioxidant properties, and these benefits were more significant when the percentage of sea buckthorn in bread increased. These changes are due to the high content in antioxidant and antimicrobial compounds of berries, such as polyphenols and carotenoids. The main polyphenolic molecules identified in the used berry flour were catechin, hyperoside, chlorogenic acid, cis- and trans-resveratrol, ferulic and protocatechuic acids, procyanidins B2 and B1, epicatechin, gallic acid, quercetin, *p*- and *m*-hydroxybenzoic acids. The bread’s organoleptic characteristics were also improved, but only for 1% added berry flour. As a consequence, the results of the present study recommend the addition of 1% flour of *Hippophae rhamnoides* berries in wheat bread, in order to obtain a product enriched in health-promoting biomolecules, with better sensorial properties and longer shelf life. It could also promote the idea of testing the addition of sea buckthorn berry flour in other bakery products.

## Figures and Tables

**Table 1:** Physicochemical indicators of the wheat flour used in bread making.

Indicator	Value
Moisture content, %	13.3 ± 0.1
Ash content, %	0.520 ± 0.001
Wet gluten content, %	25.56 ± 0.2
Acidity, degrees	2.1 ± 0.1

**Table 2:** General characteristics of the sea buckthorn berry flour used for experiments (the results are expressed as means ± standard deviations of three experiments).

Sea Buckthorn Berry Flour Characteristics	Value
Moisture content, %	7.80 ± 0.20
Ascorbic acid, mg/100 g	352.5 ± 23.4
Total polyphenols (Folin-Ciocalteu), mg GAE/100 g	1467 ± 471
Total polyphenols (Abs280), mg GAE/100 g	1311 ± 105
Total flavonoids, mg GAE/100 g	555 ± 61
Cinnamic acids, mg CAE/100 g	425 ± 34
Flavonols, mg QE/100 g	668 ± 33
Total carotenoids, mg/100 g	34.93 ± 1.30
ABTS Antioxidant activity, mmol TE/100 g	7.64 ± 0.41
DPPH Antioxidant activity, %	67.99 ± 1.20
Catechin, mg/100 g	35.3 ± 5.1
Hyperoside, mg/100 g	23.6 ± 12.1
Chlorogenic acid, mg/100 g	11.1 ± 6.3
Cis-resveratrol, mg/100 g	10.8 ± 7.5
Trans-resveratrol, mg/100 g	10.4 ± 0.4
Ferulic acid, mg/100 g	10.3 ± 1.6
Protocatechuic acid, mg/100 g	7.0 ± 0.9
Procyanidin B2, mg/100 g	4.3 ± 1.7
Epicatechin, mg/100 g	2.5 ± 1.8
Gallic acid, mg/100 g	2.2 ± 0.5
Procyanidin B1, mg/100 g	1.6 ± 0.2
Quercetin, mg/100 g	0.9 ± 0.8
***p***-hydroxybenzoic acid, mg/100 g	0.8 ± 0.2
Syringic acid, mg/100 g	0.7 ± 0.3
***m***-hydroxybenzoic acid, mg/100 g	0.5 ± 0.1
Vanillic acid, mg/100 g	0.5 ± 0.2
***p***-coumaric acid, mg/100 g	0.3 ± 0.2
Caffeic acid, mg/100 g	0.2 ± 0.0
Sinapic acid, mg/100 g	nd
Polydatine, mg/100 g	nd
Salicylic acid, mg/100 g	nd
Ferulic acid methyl ester, mg/100 g	nd
Gentisic acid, mg/100 g	nd

nd = not detected.

**Table 3:** Sensory profiles of bread samples prepared with sea buckthorn berry flour (different letters designate statistically different results).

	Control	1% SBBF	3% SBBF	5% SBBF
Product shape and volume	4.00 ± 0.00 ^c^	4.00 ± 0.00 ^c^	3.34 ± 0.01 ^b^	3.20 ± 0.05 ^a^
Crust appearance and color	4.00 ± 0.00 ^c^	4.00 ± 0.00 ^c^	3.72 ± 0.02 ^b^	3.58 ± 0.03 ^a^
Baking degree, state and appearance of bread core	5.80 ± 0.02 ^b^	6.00 ± 0.00 ^c^	6.00 ± 0.00 ^c^	5.60 ± 0.04 ^a^
Bread core porosity and pore structure	6.00 ± 0.00 ^c^	6.00 ± 0.00 ^c^	5.30 ± 0.06 ^b^	5.10 ± 0.07 ^a^
Aroma	4.00 ± 0.00 ^c^	4.00 ± 0.00 ^c^	3.60 ± 0.04 ^b^	3.20 ± 0.07 ^a^
Taste	6.00 ± 0.00 ^c^	6.00 ± 0.00 ^c^	5.40 ± 0.03 ^b^	5.20 ± 0.05 ^a^
Total score on organoleptic	29.80 ± 0.20 ^c^	30.00 ± 0.00 ^c^	27.36 ± 0.24 ^b^	25.88 ± 0.31 ^a^

**Table 4:** Influence of different quantities of sea buckthorn berry flour (SBBF) addition on wheat bread quality (errors represent the standard deviations of three replicates and different letters designate statistically different results).

Bread Quality Parameters	Control	1% SBBF	3% SBBF	5% SBBF
Moisture content, %	42.0 ± 0.32 ^a^	42.5 ± 0.28 ^a^	43.2 ± 0.30 ^b^	43.7 ± 0.25 ^b^
Acidity, degrees.	1.2 ± 0.1 ^a^	2.4 ± 0.2 ^b^	3.2 ± 0.2 ^c^	4.7 ± 0.2d
Porosity, %	72.3 ± 1.4 ^a^	72.7 ± 1.3 ^a^	68.2 ± 1.2 ^b^	59.7 ± 1.5 ^c^
Bread specific volume, cm^3^/100 g	237 ± 18 ^a^	247 ± 14 ^a^	195 ± 12 ^b^	181 ± 10 ^b^

**Table 5:** The influence of sea buckthorn berry flour on the development of rope spoilage during wheat bread storage.

Storage Time of Wheat Bread Samples Before Appearance of Rope Spoilage, Hours	Control	1.0% SBBF	3.0% SBBF	5.0% SBBF
24	−	−	−	−
48	−	−	−	−
72	+	−	−	−
96	++	+	−	−
120	+++	+++	+	−
144	+++	+++	++	+

“−”—no signs of microbial alteration. “+”—early signs of rope spoilage development “++”—moderate development of rope spoilage (stickiness and unpleasant odor) “+++”—advanced rope spoilage (increased stickiness and unpleasant odor).

**Table 6:** Criteria for sensory evaluation of wheat bread.

Sensory Characteristic	Scale	Product Description	Points
Shape and volume	0...4	Correct shape, symmetrical, aesthetic. Volume well developed. Non-flattened or bulged.	4
		Correct shape, but asymmetrical. Volume well developed.	2
		Deformed shape.	0
Crust appearance and color	0...4	Beautiful yellowish crust for white bread. Uniform color; smooth, glossy crust surface, without defects. Crispy crust.	4
		Uniformly browned crust, too dark or too pale parts, rough surface. Cracks less than 1 cm wide and less than 5 cm long. Not crispy and slightly soft crust.	2
		Whitish crust or too browned parts larger than ¼ of the wrinkled surface or dirty crust. Cracks 1 cm wide and 5 cm long.	0
Baking degree, state and appearance of bread core	0...6	Well baked. Very elastic core, uniform color, not crumbly.	6
		Sufficiently baked. Soft crust. Medium elastic core, not crumbly.	3
		Unbaked dough residues. Not elastic core, crumbly.	0
Bread core porosity and pore structure	0...6	Uniform core porosity, fine (fluffy) pore structure, maximum two holes of maximum 1 cm^2^ are present in section.	6
		Uniform core porosity, fine (fluffy) pore structure, pores of maximum size 1 cm^2^ are present in section.	4
		Uneven core porosity, maximum four gaps of approx. 2 cm^2^ are present in section.	2
		Large holes in the section and very low porosity.	0
Aroma	0...4	Pleasant pronounced aroma of well fermented and well baked bread.	4
		Mild aroma, without any foreign notes.	2
		No aroma. Foreign odors are present.	0
Taste	0...6	Pleasant, mildly sour-sweet, characteristic for every assortment	6
		Relatively good, satisfactory taste.	4
		Slightly unpleasant, sour, flat or salty taste	2
		Pronounced sour, flat or salty taste.	0
